# The Search for Associations of Serum Proteins with the Presence of Unstable Atherosclerotic Plaque in Coronary Atherosclerosis

**DOI:** 10.3390/ijms232112795

**Published:** 2022-10-24

**Authors:** Ekaterina Mikhailovna Stakhneva, Elena Vladimirovna Kashtanova, Yana Vladimirovna Polonskaya, Eugeniia Vitalievna Striukova, Viktoriya Sergeevna Shramko, Evgeny Viktorovich Sadovski, Alexey Vitalievich Kurguzov, Ivan Sergeevich Murashov, Alexander Mikhailovich Chernyavskii, Yuliya Igorevna Ragino

**Affiliations:** 1 Research Institute of Internal and Preventive Medicine—Branch of the Institute of Cytology and Genetics, Siberian Branch of Russian Academy of Sciences, 630089 Novosibirsk, Russia; 2 The Federal State Budgetary Institution “National Medical Research Center named academician E.N. Meshalkin” of the Ministry of Health of the Russian Federation, 630055 Novosibirsk, Russia

**Keywords:** proteomics, quantitative proteomics, coronary atherosclerosis, ceruloplasmin, haptoglobin

## Abstract

To study the associations of blood proteins with the presence of unstable atherosclerotic plaques in the arteries of patients with coronary atherosclerosis using quantitative proteomics. The studies involved two groups of men with coronary atherosclerosis (group 1 (St) had only stable atherosclerotic plaques; group 2 (Ns) had only unstable atherosclerotic plaques, according to histological analysis of tissue samples); the average age of patients was 57.95 ± 7.22. Protein concentrations in serum samples were determined using the PeptiQuant Plus Proteomics Kit. The identification of protein fractions was carried out by monitoring multiple reactions on a Q-TRAP 6500 mass spectrometer combined with a liquid chromatograph. Mass spectrometric identification revealed in serum samples from patients with unstable atherosclerotic plaques a reduced concentration of proteins in the blood: α-1-acid glycoprotein, α-1-antichymotrypsin, α-1-antitrypsin, ceruloplasmin, hemopexin, haptoglobin, apolipoprotein B-100, apolipoprotein L1, afamin and complement component (C3, C7, C9). Moreover, at the same time a high concentration complements factor H and attractin. The differences were considered significant at *p* < 0.05. It was found that the instability of atherosclerotic plaques is associated with the concentration of proteins: afamin, attractin, components of the complement system, hemopexin and haptoglobin. The data of our study showed the association of some blood proteins with the instability of atherosclerotic plaques in coronary atherosclerosis. Their potential role in the development of this disease and the possibility of using the studied proteins as biomarkers requires further research.

## 1. Introduction

Research in the field of etiology and pathogenesis of coronary atherosclerosis is currently relevant due to the high prevalence and mortality of this disease. Atherosclerosis is the basis of cardiovascular diseases (CVD) and includes a number of pathological processes, such as endothelial dysfunction, extensive deposition of lipids in the intima, exacerbations of congenital and adaptive immune reactions, proliferation of smooth muscle cells and remodeling of the extracellular matrix, eventually leading to the formation of atherosclerotic plaque.

Atherosclerotic lesions have a high degree of heterogeneity of their composition, which can affect the progression of the pathological atherosclerotic process, leading to the development of complications of CVD. For the early diagnosis of cardiovascular diseases of atherosclerotic genesis, acute phase proteins and proteins involved in the implementation of the immune response of the body are of great importance. Ceruloplasmin is an acute phase protein that increases in inflammatory diseases. Elevated concentrations of both ceruloplasmin and copper are associated with an increased risk of mortality from all causes and from cardiovascular causes [1]. Many studies show the activation of the complement system in atherosclerosis [2,3]. Experimental data confirm the role of complement system proteins at all stages of arterial hypertension [4]. The detection of deposits of a large amount of C3b/iC3b and MAC in arterial samples of patients with atherosclerosis indicates complement activation. Excessive complement activation plays an important role in the pathogenesis of atherosclerosis [5]. The level of haptoglobin significantly reduced in patients with stable coronary artery disease (CHD) and acute coronary syndrome (ACS) compared with healthy controls. Logistic regression analysis showed that a decrease in haptoglobin expression correlates with an increased risk of coronary heart disease [6]. Patients with acute myocardial infarction (MI) have elevated concentrations of alpha-1-acid glycoprotein [7].

The accumulation of knowledge about the contribution of specific proteins to the pathogenesis of coronary atherosclerosis and its complications, together with the development of modern research methods, contributes to the search for proteins-potential prognostic and diagnostic biomarkers of CVD. Quantitative proteomic analysis, used for the identification and determination of biological molecules based on mass spectrometry with tandem chemical labels, is a method with accurate quantitative simultaneous determination of a large number of proteins in various biological samples. Understanding changes in the protein component of blood in atherosclerosis will help to identify new biomarkers for a better understanding of the conditions for the development of complications of this disease.

The aim of this research was to study the association of certain blood proteins with the presence of unstable atherosclerotic plaques in the arteries of men with coronary atherosclerosis using quantitative proteomic analysis.

## 2. Results

Proteomic profiling of blood serum samples was performed using the PeptiQuant Plus Proteomics Kit. We identified 125 proteins. The identification of proteins was carried out by monitoring multiple reactions on a mass spectrometer with electrospray ionization combined with a high-performance liquid chromatograph.

The analysis of the differential expression of the protein was carried out by two technical repeats of each sample. After comparative analysis, proteins were isolated, the concentration of which was statistically significantly different between group 1 (St) and group 2 (Ns) (*p* < 0.05).

The concentration of the main blood protein, serum albumin, did not differ in the study groups (Table 1). Acute phase proteins studied for a very long time and in connection with inflammatory diseases. In addition to the well-known C-reactive protein, these are serum glycoproteins: α-1-acid glycoprotein, α-1-antichymotrypsin and α-1-antitrypsin. Previously showed that α-1-antitrypsin, 1-acid glycoprotein, ceruloplasmin and CRP are associated with the severity of coronary atherosclerosis [8]. A-1-acid glycoprotein had in elevated concentrations in patients with acute myocardial infarction and had anti-inflammatory properties [7]. In our study, the blood concentrations of acute phase inflammatory proteins (α-1-acid glycoprotein, α-1-antichymotrypsin and α-1-antitrypsin) differ significantly (Table 1).

For the early diagnosis of cardiovascular diseases of atherosclerotic genesis, acute phase proteins and proteins involved in the implementation of the immune response of the body are of great importance. Ceruloplasmin is an acute phase protein that increases in inflammatory diseases. Elevated concentrations of both ceruloplasmin and copper are independently associated with an increased risk of mortality from all causes and from cardiovascular causes [1]. In our study, the concentration of ceruloplasmin in the blood in the group of patients with unstable plaques was 13% lower (*p* < 0.05) (Table 1).

Haptoglobin is of interest from the group of acute phase proteins. It is known that its plasma level is inversely associated to many inflammatory diseases, including CVD [9]. Haptoglobin forms a hemoglobin-haptoglobin complex involved in iron homeostasis in the body. In the study groups, the content of haptoglobin in the blood significantly differed, in the group of patients with unstable plaques it was 19% less than in group 1 (St) (Table 1). At the same time, the concentration of the alpha protein of the hemoglobin subunit in the blood decreased in group 2 (Ns) but did not reach statistical significance.

Another protein that participates in iron homeostasis is hemopexin. Hemopexin binds hemoglobin and free heme of erythrocytes and suppresses excessive oxidative activity. The concentration of this protein increases several times during inflammation, and it is also referred to as acute phase glycoproteins [10,11]. The concentration of hemopexin protein in the blood significantly differed in the study groups, and in group 2 (Ns) it was 11% less than in group 1 (St) (Table 1). A multifactorial logistic analysis showed that the instability of atherosclerotic plaques is associated with the concentration of hemopexin and haptoglobin (Table 2).

It is known that plaques of male patients have higher levels of ferritin and transferrin receptor 1 in the lesions, but low levels of serotransferrin and hemopexin in comparison with plaques of female patients [12]. In our study, the concentration of serotransferrin in patients was 9% lower than in the St group (Table 1).

Participating in the homeostasis of iron and copper, phase proteins are not only acute phase proteins but also transport proteins. Of the transport proteins, retinol-binding protein 4 (RBP4) and transthyretin are of interest, which are functionally interacting proteins that form a transport complex with vitamin A. In our study, the concentration of RBP4 was 10%, and the level of transthyretin was 25% higher in group 2 (Ns) patients than in group 1 (St) (Table 1) patients, but no statistical significance was achieved. However, multifactorial logistic analysis showed that the instability of atherosclerotic plaques is associated with the concentration of RBP4 (Table 2).

It was seen that afamin can play the role of a carrier of vitamin E in plasma and other bodily fluids under physiological conditions. The study demonstrated the specific affinity of afamin for binding to both α-tocopherol and γ-tocopherol, the two most important forms of vitamin E [13]. In our study, the concentration of afamin was 20% lower in group 2 (Ns) patients than in group St (Table 1).

Dysfunction of lipid metabolism is the main risk factor for cardiovascular diseases of atherosclerotic genesis. The key regulators of lipoprotein metabolism are apolipoproteins (apo). In our study, the concentrations of apolipoproteins A and C in the study groups did not differ. Apo B-100 and apo L1 significantly increased in group 2 (Ns), by 24% and 25%, respectively (Table 1).

In addition to acute phase proteins and transport proteins, proteins involved in the implementation of the immune response are important for the diagnosis of the disease. Sequential reactions of activation of components of the complement system (C1, C2, C3, C4, C5, C6, C7, C9) take part in the development of the inflammatory process. In our study, there was no significant difference between the C1 (C1q; C1r; C1s) subcomponents in the study groups (Table 1). However, the total content of C1 complement subcomponents was higher in group 1 (St) (471.37 fmol/µL) than in group 2 (Ns) (446.48 fmol/µL), *p* < 0.0001.The content of complement components C3, C7, C9 and complement factor B was higher in patients of group 1 (St) (*p* < 0.05). The level of complement factor H involved in inactivation of C3b was 10% higher in patients of group 2 (Ns) (Table 1). In addition, multifactorial logistic analysis showed that the instability of atherosclerotic plaques is associated with the concentration of complement components (Table 2).

Attractin is a protein that is expressed in human blood monocytes and released by activated T-cells. The attractin is involved in the initial clustering of immune T cells during an inflammatory reaction [14]. The attractin content in the blood was 14% higher in group 2 (Ns) (Table 1), and multivariate logistic analysis showed an association between the instability of atherosclerotic plaques and the concentration of attractin (Table 2).

## 3. Discussion

Human serum albumin is the main protein of human blood, performs a transport function, and has antioxidant activity due to its ability to bind reactive oxygen species [15]. Human serum albumin is able to bind many ligands, including proatherogenic, thereby preventing their contribution to oxidative stress [16]. It demonstrated that a low concentration of serum albumin in the blood is a prognostic factor of atherosclerosis in blood vessels, regardless of traditional risk factors and statin therapy in patients with HIV infection [16]. In our study, the concentration of serum albumin in the groups of patients with stable and unstable atherosclerotic plaques did not differ.

Earlier, when studying sexual dimorphism in carotid atherosclerotic plaques, it showed that the content of acute phase proteins alpha-1-acid glycoprotein 2 and alpha-1-antichymotrypsin significantly increased in lesions in women compared with men, with a specific increased content in the areas of internal control and the center of the plaque, respectively [17]. In our study, the blood concentrations of proteins, α-1-acid glycoprotein, α-1-antichymotrypsin and α-1-antitrypsin, differed significantly in the groups. The concentration of these proteins in group 2 (Ns) was significantly lower. This fact is consistent with the earlier conclusion of Wagsater et al. that the vascular expression of α-1-antichymotrypsin is associated with human vascular diseases, atherosclerosis of the carotid arteries, and abdominal aortic aneurysm. It assumed that α-1-antichymotrypsin contributes to the stability of plaques [18].

Ceruloplasmin is a specific copper-containing plasma glycoprotein that belongs to the acute phase proteins and has pro- and anti-inflammatory properties, therefore its role in atherosclerosis is contradictory. Most of the studies confirm a direct link between elevated ceruloplasmin levels and the frequency of coronary heart disease [19]. It was shown that a high level of ceruloplasmin in serum was associated with a higher risk of myocardial infarction after 3 years (HR 2.35, 95% CI 1.79-3.09, *p* < 0.001). After adjusting for traditional risk factors, highly sensitive C-reactive protein and creatinine clearance ceruloplasmin remained an independent predictor of adverse events (HR 1.55, 95% CI 1.10–2.17, *p* = 0.012) [20]. Nevertheless, in our case-control study, we revealed a decrease in the level of some isoforms of ceruloplasmin in patients with coronary artery disease and coronary atherosclerosis [21]. In this study, when comparing groups with stable and unstable plaques, we found a decrease in ceruloplasmin in the blood in group 2 (Ns).

Haptoglobin is a circulating acute phase protein produced by the liver and adipose tissue, induced in prooxidant conditions, such as systemic inflammation or obesity [22]. It is known that its plasma level is inversely associated with many inflammatory diseases, including cardiovascular diseases. It showed that the genetic variant of haptoglobin is not associated with the severity of coronary heart disease and mortality in the general population. However, hypertensive patients with the allele T rs217181, and with a higher level of haptoglobin, had more severe coronary heart disease in the Chinese population [9]. Haptoglobin participates in iron homeostasis in the body, forming the hemoglobin-haptoglobin complex. In the studied groups, the content of haptoglobin in the blood was significantly different, while the concentration of hemoglobin subunit alpha protein in the blood in patients in group 2 (Ns) decreased but did not reach statistical significance. Although, it is known that male patients in atherosclerotic plaques have a large foci of necrosis and a large number of plaque ruptures, which is associated with a higher level of hemoglobin in the blood [12]. It is known that iron accumulates in atherosclerotic plaques and affected areas of the arteries and in a catalytically active form can cause proatherogenic events, such as the production of reactive oxygen species and lipid peroxidation [23]. Serotransferrin is an iron transport protein. When studying gender differences in atherosclerotic plaques, it was found that the atheromas of male patients have higher levels of ferritin and transferrin receptor 1 in the plaques but low levels of serotransferrin and hemopexin in comparison with the plaques of female patients [12]. In our study, the concentration of circulating serotransferrin in Ns patients was 9% lower than in the group St, which is consistent with lower hemoglobin levels in group 2 (Ns).

The protein hemopexin is a participant in iron homeostasis that is capable of binding hemoglobin, protecting from possible oxidative damage. The concentration of this protein increases with inflammation and its acute phase glycoprotein [10]. Therefore, heme-binding hemopexin is considered by some researchers as a protective protein in this process, although its role in atherosclerosis has not been fully elucidated [24]. When comparing groups with stable and unstable plaques, the decrease in the levels of acute phase proteins, ceruloplasmin, hemopexin and haptoglobin in the blood in group 2 (Ns) can explained by the fact that inflammatory processes more pronounced in patients with stable fibrous plaques.

Participating in the homeostasis of iron and copper, these proteins are not only acute phase proteins but also transport proteins.

Of the transport proteins, RBP4 and transthyretin are of interest, which are functionally interacting proteins that form a transport complex with vitamin A. It showed that there is a correlation between the concentration of free RBP and the thickness of intima media, a diagnostic indicator of carotid artery atherosclerosis [25]. In addition, the concentration of RBP4 in the blood is associated with cardiovascular risk factors associated with insulin resistance and CHD, circulating RBP4 can be a marker of metabolic complications and atherosclerosis and used to assess CHD [26,27]. In early studies, we found an increase in the levels of transthyretin transport proteins and RBP4 in the blood of patients with coronary atherosclerosis compared with controls [28]. Transthyretin-amyloidosis occurs due to the accumulation of transthyretin-amyloid in the extracellular space of various organs and systems, especially the heart and nervous system [29]. The Cubedo et al. study was the first to show the relationship between the concentrations of different forms of transthyretin in the blood with the risk of developing cardiovascular diseases [30]. In our study, despite the lack of statistical significance, the levels of circulating RBP4 and transthyretin were 10% and 25% higher, respectively, in patients in group 2 (Ns).

The glycoprotein afamin is a member of the albumin family. Previously, proteomic studies identified afamin as a potential biomarker of ovarian cancer. In patients with ovarian cancer, there was a significant decrease in the concentration of afamin in the blood compared to the control group [13]. Additionally, by proteomic analysis, afamin was determined as a predictor of gestational diabetes mellitus in pregnant women [31,32]. Nevertheless, in a pilot study performed using tandem mass spectrometry, a significant increase in the level of afamin was found in samples of carotid atherosclerotic plaques [17]. In addition, the role of afamin as a transport protein, a carrier of vitamin E, a well-known antioxidant, in plasma and other body fluids was shown [13]. In our study, the concentration of afamin was significantly lower in group 2 (Ns). Given the specific affinity of afamin to tocopherol, it can assumed that the decrease in protein is associated with the concentration of the antioxidant in the blood. Thus, the role of afamin in the pathophysiology of the atherosclerotic process is to be clarified in future studies.

Dysfunction of lipid metabolism is the main risk factor for CVD. The key regulators of lipoprotein metabolism are transport proteins apolipoproteins. Despite the fact that a large number of apolipoproteins are known in clinical practice as methods for determining ApoB and ApoA-I in the blood and are used to assess cardiovascular risk. In the LIFE-Heart study, when assessing the diagnostic potential of apolipoproteins in the blood by LC-MS/MS, it showed that apolipoproteins A-IV, B-100, C-III and E are independently associated with atherosclerotic CVD, and ApoC-III and apoE are independently associated with plaques in the carotid arteries [33]. In our study, we found no differences in the concentrations of apolipoproteins A and C in the study groups. However, apo B-100 and apo L1 increased significantly in the group 2 (Ns).

In addition to acute phase proteins and transport proteins, proteins involved in the implementation of the immune response are important for the diagnosis of atherosclerotic disease. The complement system includes approximately 20 interacting proteins involved in the implementation of the immune response and the development of the inflammatory process. Activation of the complement system is associated with atherosclerosis and CVD [34,35]. Complement system proteins are associated with vascular remodeling and atherosclerosis [36]. Research data indicate that the anomaly of complement components and the resulting excessive activation of complement are associated with atherogenesis. Detection of C3b/iC3b and membrane-attacking complex deposits in atherosclerotic arteries indicates increased complement activation [5]. High levels of C5b-9 was found in intimal thickening and fibrous plaques compared to normal tissue. At the same time, the levels of C5b-9 in intimal thickenings were higher than in fibrous plaques, which allowed the authors to assume that complement activation occurs directly in the artery wall [2].

Wezel et al. showed that the complement component C5a and its receptor C5aR was expressed in vulnerable atherosclerotic plaques. A significant increase in C5aR in the plaque was found in mice treated with C5a, while local treatment with C5a led to increased plaque destruction with hemorrhage [3]. In another study, it was found that the membrane-attacking complex can play a crucial role in the formation of plaques and the rupture of aneurysms [37]. In our study, there was no significant difference between the C1 (C1q; C1r; C1s) subcomponents in the studied groups. In addition, we did not receive an increase in the concentration of complement components C7 and C9 involved in the formation of a membrane-attacking complex in the blood of patients with unstable plaques. Apparently, this process is exclusively local in the tissue of the atherosclerotic plaque.

However, in the blood of two group (Ns), we identified a high level of attractin, a protein also involved in the implementation of the immune response. In addition, the level of attractin in the blood was proposed to be used to predict the slow or rapid growth of abdominal aortic aneurysms in humans, because the level of attractin in the blood significantly correlates with the future growth of abdominal aortic aneurysms [38].

## 4. Materials and Methods

The study included patients with coronary artery disease and coronary atherosclerosis who were admitted for coronary bypass surgery and underwent endarterectomy from the coronary arteries during the operation according to intraoperative indications. Exclusion criteria: myocardial infarction less than 6 months, acute chronic infectious and inflammatory diseases or their exacerbations, renal failure, active liver diseases, cancer, hyperparathyroidism. The Ethics Committee of IIPM–Branch of IC&G SB RAS (Protocol № 7 of 26.09.2017) approved the protocol of the study. All participants signed an Informed Consent to participate in the study. The study material is blood serum samples. The blood was taken from the vein in the morning on an empty stomach in all patients.

The serum samples of 40 men were selected for quantitative proteomic analysis. All patient samples were divided into 2 groups. The first group consisted of 20 patients who, according to histological analysis, had only stable atherosclerotic plaques (AP) in the samples of AP; the average age of patients was 58.50 ± 4.25 and BMI 28.76 ± 4.44. The second group consisted of 20 patients who, according to histological analysis, had only un-stable atherosclerotic plaques in the samples of AP; the average age was 57.40 ± 9.79 and BMI was 29.33 ± 5.04.

### 4.1. Sample Preparation

Determination of protein concentration in serum samples was carried out using the PeptiQuant Plus Proteomics Kit (Cambridge Isotope Laboratories, Tewksbury, MA, USA), according to the manufacturers’ method and with some modifications.

A total of 20 mL of a solution containing 9 M urea, 20mM dithiothreitol, 300 mM Tris-HCl (pH 8.0) was added to 10 mL of a serum sample for performed trypsinolysis in solution. In a separate test tube, 10 µL of a solution of bovine serum albumin (BSA) was added, which was later used as a matrix solution for calibration points. The samples were incubated for 30 min at 37 °C. The 20 µL of 100 mM iodoacetamide solution was added to all test tubes and incubated for 30 min in the dark at room temperature. Later, 272 µL of 100 mM Tris-HCl (pH 8.0) and 35 µL of trypsin solution were added and incubated for 18 h at 37 °C. Proteolysis was stopped by adding 343 µL of 2% formic acid and incubated for 18 h at 37 °C. Proteolysis was stopped by adding 343 µL of 2% formic acid.

A mixture of light (unlabeled) peptides was diluted in 60 µL of a solution of 30% ace-tonitrile and 0.1% formic acid. We prepared a series of dilutions for calibration according to the plan. A mixture of peptides labeled with heavy stable isotopes was diluted in 450 µL with 30% acetonitrile and 0.1% formic acid and used as an internal standard.

A total of 40 µL of serum samples was added to the test tubes after trypsinolysis and 40 µL of BSA solution was added after trypsinolysis. A total of 10 µL of a solution of labeled peptides was added to all tubes. A total of 10 µL of the dilution of standards was added to the tubes with BSA for the calibration curves. A total of 10 µL of 30% acetonitrile and 0.1% of formic acid was added to test tubes with serum samples. A total of 540 µL of 0.1% formic acid was added to all tubes.

The samples were cleaned on Oasis HLB solid-phase extraction cartridges (Waters, Milford, MA, USA), 10 mg. The cartridges activated 600 µL of methanol and balanced 600 µL of 0.1% formic acid. A total of 510 µL of the sample was added and washed in 600 µL of water three times. Peptides were eluted with 300 µL of 50% acetonitrile, 0.1% formic acid. The obtained samples were frozen at −80 °C and dried using FreeZone 2.5 (Labconco, Kansas, MO, USA). Dry sediments were diluted in 34 µL of 0.1% formic acid and used for analysis.

### 4.2. Mass Spectrometry Analysis

The detection of peptides was carried out by the method of Multiple Reaction Monitoring (MRM) on a Q-TRAP 6500 mass spectrometer (AB Sciex, Framingham, MA, USA) combined with an high-performance liquid chromatograph Infinity 1290 (Agilent, Canta Clara, CA, USA).

Chromatographic separation was carried out on a column Titan C18, 1,9 µm, 10 cm × 2.1 mm (Supelco, Bellefonte, PA, USA) in several stages: 0–40 min–from 2% to 23% of phase B, 40–43 min–from 23% to 45%, 43–43.5 min–from 45% to 80%, 43.5–45.5 min–80% of phase B, 45.5–46 min–from 80% to 2%, 46–50 min 2% of phase B. Mobile phase A: 99.9%–water, 0.1%–formic acid; phase B: 99.9%–acetonitrile, 0.1%–formic acid. The flow rate was 0.4 mL/min, the separation temperature is 45 °C. Positively charged ions obtained by electrospray ionization in the Turbo Spray IonDrive source were detected. The source temperature was 400 °C, the capillary voltage was 4 kV, the curtain gas pressure was 40 psi, the gas pressure was40 psi, and the decasterization potential was 40 V.

Calibration curves were constructed and protein concentrations were determined using the MultiQuant 3.0.2 program (AB Sciex, Framingham, MA, USA) based on the area of peaks of MRM transitions specific to each studied peptide.

### 4.3. Statistical Analysis

Statistical data processing was carried out using the SPSS 23.0 for Windows. Statistical analysis included a test for the normality of Kolmogorov–Smirnov traits, a comparative analysis of One Way Anova between groups using the Student’s t-test for normally distributed variables, or a Mann–Whitney comparative analysis for abnormally distributed variables. To improve the perception of data, the results in the table are given in the form of average values and standard quadratic deviation of variables (Mean ± SD). In order to search for associations, a multifactorial logistic regression analysis was carried out, where the variable “stable/unstable plaques” used as a dependent variable, and the studied proteins were included in small groups as independent variables. The differences were considered statistically significant at *p* < 0.05.

## 5. Conclusions

The prognostic value of the studied proteins as biomarkers of atherosclerotic plaque instability in coronary atherosclerosis requires further research.

The results of this study, obtained using the modern tandem mass spectrometry method, revealed elevated concentrations of attractin proteins and the complement subcomponent factor H in blood from patients with unstable atherosclerotic plaque. Moreover, a reduced content of proteins involved in the implementation of the immune response and the development of the inflammatory process: α-1-acid glycoprotein, α-1-antichymotrypsin, α-1-antitrypsin, ceruloplasmin, hemopexin, haptoglobin, apolipoprotein B-100, apolipoprotein L1, afamin and complement component (C3, C7, C9).

Multivariate logistic regression analysis confirmed the association of instability with the concentration of afamin (Exp(B) = 0.988; *p* = 0.001), hemopexin (Exp(B) = 0.997; *p* = 0.020), haptoglobin (Exp(B) = 0.967; *p* = 0.001) and complement component. It is possible that the increased concentration of these proteins in the blood will be considered as a promising potential biomarker of atherosclerotic plaque instability in coronary atherosclerosis in the future. This study presents a potential proteomic platform for further investigations into plaque instability in atherosclerosis.

Study limitation: The number of persons included in the study is small (40 people). The absence of a healthy control group is a study limitation. The design of the study was to examine only the associations of serum proteins with the presence of unstable atherosclerotic plaques in the arteries of patients (men) with coronary atherosclerosis in comparison with a group of patients with stable lesions.

## Figures and Tables

**Table 1:** Quantitative mass spectrometric identification of proteins in the blood (Mean ± SD).

№	Protein Name	Protein Concentration, fmol/µL		*p*
		Group 1 (St)	Group 2 (Ns)	
1	Ceruloplasmin	1891.77 ± 511.66	1646.48 ± 418.60	0.021
2	Serum albumin	374,440.00 ± 61,793.83	354,465.00 ± 58,076.57	0.140
3	α-1-acid glycoprotein	18,027.10 ± 7298.18	13,287.65 ± 4678.42	0.001
4	α-1-antichymotrypsin	6224.75 ± 3299.37	4545.25 ± 2367.32	0.011
5	α-1-antitrypsin	27,696.0 ± 7929.29	23,672.0 ± 9887.34	0.048
6	Hemoglobin subunit α	4785.9 ± 2342.02	4204.15 ± 2608.95	0.297
7	Haptoglobin	589.55 ± 261.55	479.60 ± 194.18	0.036
8	Hemopexin	1973.6 ± 247.48	1756.55 ± 310.65	0.001
9	Serotransferrin	19,999.50 ± 3002.74	18,329.50 ± 3243.45	0.019
10	Retinol-binding protein 4	1237.08 ± 287.36	1372.42 ± 413.33	0.093
11	Transthyretin	510.13 ± 179.43	640.23 ± 456.87	0.098
12	Afamin	330.12 ± 117.85	264.59 ± 73.53	0.004
13	Apolipoprotein A-I	21,096.25 ± 6127.08	21,626.0 ± 3662.74	0.640
14	Apolipoprotein B-100	276.24 ± 79.53	211.04 ± 68.84	0.0001
15	Apolipoprotein C-I	5019.2 ± 1251.40	5069.6 ± 1353.80	0.863
16	Apolipoprotein L1	590.28 ± 158.45	501.25 ± 200.51	0.031
17	Complement C1q subcomponent subunit B	75.86 ± 31.96	67.07 ± 17.17	0.129
18	Complement C1q subcomponent subunit C	117.84 ± 36.25	120.22 ± 35.42	0.768
19	Complement C1r subcomponent	230.49 ± 51.37	210.20 ± 70.84	0.147
20	Complement C1s subcomponent	47.18 ± 10.83	48.99 ± 22.84	0.652
21	Complement C3	590.51 ± 137.97	516.46 ± 139.39	0.019
22	Complement component C7	73.23 ± 19.38	61.94 ± 11.18	0.002
23	Complement component C9	167.05 ± 66.10	138.93 ± 56.85	0.045
24	Complement factor B	4951.7 ± 1358.16	4215.7 ± 1135.39	0.010
25	Complement factor H	530.54 ± 79.29	577.37 ± 84.59	0.014
26	Attractin	48.43 ± 9.97	55.17 ± 17.14	0.035

Note: St—the group of patients who, according to histological analysis, had only stable atherosclerotic plaques in the tissue samples; Ns—the group of patients who, according to histological analysis, had only unstable atherosclerotic plaques in the tissue samples.

**Table 2:** Results of multivariate regression analysis of the association of proteins with the instability of atherosclerotic plaques (dependent variable–instability).

Proteins	B	Exp B	95.0% C.I. for Exp B	*p*
Complement component C3	0.014	1.014	1.003–1.026	0.016
Complement component C7	−0.054	0.948	0.911–0.985	0.007
Complement component C9	0.038	1.039	1.004–1.076	0.030
Complement factor H	0.030	1.031	1.008–1.055	0.009
Afamin	−0.012	0.988	0.981–0.994	0.0001
Attractin	0.044	1.045	1.005–1.086	0.027
Retinol-binding protein 4	0.011	1.011	1.004–1.017	0.001
Hemopexin	−0.003	0.997	0.995–1.000	0.020
Haptoglobin	−0.034	0.967	0.947–0.986	0.001

Note: B, unstandardized regression coefficients; Exp B: the odds ratio for each predictor variable; C.I., confidence interval; *p*, the *p* value for each predictor variable.

## Data Availability

The datasets before and after analysis in this study are available from the corresponding author on reasonable request.
